# Isolation, identification and characterization of nitrogen fixing endophytic bacteria and their effects on cassava production

**DOI:** 10.7717/peerj.12677

**Published:** 2022-01-25

**Authors:** Xiao Zhang, Juanjuan Tong, Mengmeng Dong, Kashif Akhtar, Bing He

**Affiliations:** 1 College of Agriculture, Guangxi University, Guangxi, Nanning, China; 2 College of Life Science and Technology, Guangxi University, Guangxi, Nanning, China

**Keywords:** Cassava, Endophytic bacteria, Growth promotion, Microbacteriaceae Curtobacterium, Nitrogen fixation

## Abstract

**Background:**

Cassava (*Manibot esculenta* Crantz) is one of the most important among tuber crops. The amount of nitrogen fertilizer used for cassava production is relatively high (400 kg ha^−1^), but there are few studies on biological nitrogen fixation in this crop. Therefore, it is particularly important to study whether cassava and microorganisms have the associated nitrogen-fixing and other promoting effects of endophytic bacteria.

**Methods:**

We screened 10 endophytic bacteria using the nitrogen-free culture method from the roots of seven cassava cultivars, and the nitrogenase activity of the A02 strain was the highest 95.81 nmol mL^−1^ h^−1^. The A02 strain was confirmed as Microbacteriaceae, *Curtobacterium* using 16S rRNA sequence alignment. The biological and morphological characteristics of strain A02 were further analyzed.

**Results:**

The experimental results showed that the biomass of roots, stems, and leaves of cassava inoculated with A02 increased by 17.6%, 12.6%, and 10.3%, respectively, compared to that of the control (without A02 inoculation). These results were not only related to the secretion of auxin (IAA) and solubilization of phosphate but also in the promotion of biological nitrogen fixation of cassava leaves by strain A02. Moreover, the highest 95.81 nmol mL^−1^h^−1^ of nitrogenase activity was reported in strain A02, and thus more nitrogen fixation was observed in strain A02. In conclusion, A02 is a newly discovered endophytic nitrogen-fixing bacteria in cassava that can be further used in the research of biological bacterial fertilizers.

## Introduction

The nitrogen-fixing capacity of associative nitrogen-fixing bacteria was not as high as that of rhizobium in legumes, but they could fix nitrogen symbiotically with non-legumes. Recently, more associative nitrogen-fixing bacteria, such as *Pennisetum* spp. *Herbaspirillum seropedicaea* strain (ATCC)35892, *Pseudomonas jessenii* strain CIP105274 (Rout & Chrzanowski, 2009), *Enterobacter cloacae* (Takada et al., 2019), *Herbaspirillum hiltneri* sp nov (Rothballer et al., 2006), *Pantoea sp*. (Loiret et al., 2004), *Pseudommonas, Bacillus*, *Burkholderia, Pantoea* (Xiaomei et al., 2018), *Azospirillum melinis sp* nov. (Peng et al., 2006), *Klebsiella oxytoca* (Adachi, Nakatani & Mochida, 2002), *Burkholderia vietnamiensis* (Tang et al., 2010), and *Klebsiella variicola* (Lin et al., 2019), have been found in sorghum, water yam, wheat, sugarcane, tea tropical molasses grass, and other monocotyledon Gramineae, such as sweet potatoes and oil palm plantlets.

Similar to rhizobium, associative nitrogen-fixing bacteria encode nitrogenase and utilize atmospheric nitrogen through biological nitrogen fixation (BNF) to convert N_2_ into inorganic nitrogen-containing compounds, such as ammonia (NH_3_), and improve the growth and yield of sugarcane (Matoso et al., 2021), sorghum (Rout & Chrzanowski, 2009), maize, wheat, cucumber (Yongbin et al., 2019); switchgrass (Roley et al., 2018), oil palm (Lim et al., 2018), and other plants (Ji et al., 2014; Golparyan, Azizi & Soltani, 2018; Tahir et al., 2020). *Pantoea* sp. NN08200 could provide up to 33% of the total nitrogen to sugarcane (Shi et al., 2019). *Pantoea, Pseudomonas, Rhanella, Herbaspirillum, Azospirillum, Rhizobium* (*Agrobacterium*), and *Brevundimonas* can offer 12–33% of the total nitrogen to maize (Montañez et al., 2009). *Paenibacillus beijingensis* BJ-18 can provide 12.9–20.9% of the total nitrogen to wheat and 52.2–59.2% to cucumber through biological nitrogen fixation (Yongbin et al., 2019). If a crop can receive about 30% of its total nitrogen through BNF, it can be considered an eco-friendly crop (Chaves et al., 2016). In addition to BNF, the associated nitrogen-fixing bacteria interact with plants in many ways to promote plant growth and enhance resistance. Many endophytes can secrete plant hormones, such as auxin, gibberellin, and abscisic acid, to promote plant growth (de Oliveira et al., 2020; Geries & Elsadany Abdelgawad, 2021). Plant hormones produced by *Azospirillum* are thought to be the main factors that promote plant growth (Keswani et al., 2020; Higdon et al., 2020). Other studies showed that plant hormones, such as auxin (IAA), gibberellin, and cytokinin, produced by bacteria enhanced root branching and elongation, increased root-hair density, and improved plant growth by facilitating the absorption of water and minerals from the soil (Steenhoudt & Vanderleyden, 2000; Santi, Bogusz & Franche, 2013). Nitrogen-fixing *Penibacillus*, *Microbacterium*, *Bacillus*, and *Klebsiella* spp. could also dissolve mineral elements, such as phosphorus, and increase the absorption of nutrients in rice (Ji, Gururani & Chun, 2014). Microbial endophyte consortia from Salicaceae and conifers have also been reported to help Douglas-fir (*Pseudotsuga menziesii*) and western red cedar (*Thuja plicata*) survive under extreme drought and degraded edaphic conditions (Aghai et al., 2019). Otherwise, nitrogen-fixing bacteria could affect the relative microbial abundance in the rhizosphere and influence plant growth (Bauer et al., 2012).

The cassava processing in the industry into starch is energy intensive, with thermal energy and electricity consumptions ranging from 1.6–2.5 MJ and 0.17–0.25 kWh per kg of processed starch, respectively (Sriroth et al., 2000). Notably, most cassava growing areas are characterized by limited and expensive energy supply, which limit advancements and new investments in the cassava industry. For instance, energy for cassava starch processing (CSP) constitutes 14% of production cost in Thailand (Chavalparit & Ongwandee, 2009) and 20–25% in Nigeria (Nang’ayo et al., 2005). Therefore, sustainable energy supply is essential for advancement of the cassava industry.

Cassava (*euphorbiaceae*) is also called tree potato or sweet potato. Its roots are rich in starch and are known as an “underground granary” and “the king of starch.” Cassava is one of the three largest potato crops, the sixth largest food crop in the world, and the third largest food crop in hot areas (Guira et al., 2017). Globally, the planting area has reached 1,700 hectares, which is important heat energy in the tropics and subtropics, and cassava is also a staple food crop for more than 600 million people in the world (Liang et al., 2016). Guangxi is the main planting and processing province of cassava in China, with a planting area of 3 million acres, accounting for more than 60% of the country in both area and output (Rosenthal, 2020). In addition, cassava, as a raw material of biofuel ethanol, has broad market prospects. It is always grown on under nourished and arid soils because it cannot compete with other staple food crops (rice, wheat, maize). Jiang et al. (2016) reported that 72–75 kg hm^−2^ N is sufficient for cassava growth, which is equivalent to 50% of the amount needed for rice growth (Jiang et al., 2016). Therefore, cassava is usually grown in low-fertile soils, and it is possible to achieve about 20 tons ha^−1^ of production (Purnamasari, Noguchi & Ahamed, 2019). In addition, it is hypothesized that cassava growth is related to biological nitrogen fixation by microorganisms (Teixeira et al., 2007).

N, P, and K fertilizer are the three important nutrients for cassava tuberization (Odedina, Ojeniyi &Odedina, 2010). Inorganic fertilizers usually have 10–20 times higher concentrations of these nutrients but organic fertilizer also contain many secondary- and micro-nutrients, and thus pay to higher yields (Howeler et al., 2005). Though, the nutrient uptake is highly related to soil functionality, plant growth rate, and climatic conditions (Howeler, 2002). The overuse of fertilizer significantly affected on environment (Akhtar et al., 2020), especially when the additive effect from the applied fertilizer is factored in (Howeler, 2002). Further, agronomic efficacy was increased with the combine application of organic and inorganic fertilizer, but the excessive use of fertilizer resulted in low agronomic efficiency (Vanlauwe et al., 2020).

However, there are few reports on cassava combined with nitrogen-fixing bacteria. Therefore, it is particularly important to study whether cassava and microorganisms have the associated nitrogen-fixing effect and other promoting effects of endophytic bacteria. In the present study, we aimed to isolate and screen endophytic nitrogen-fixing bacteria from the roots of cassava varieties, identify and analyze the biological characteristics and promoting effects of different strains, and to verify the nitrogen-fixing and promoting effects.

## Materials and Methods

### Cassava sample preparation and isolation of endophytic bacteria

Seven cassava cultivars (Guire 4, South China 205, Fuxuan 01, South China 124, South China 8, KU50, and South China 10) were planted at Hengxian Cassava Planting Base, Nanning, China. The root samples (<5 cm length and 2–3 mm diameter) were collected for the isolation of nitrogen-fixing bacteria. Roots were surface sterilized with 70% ethanol for 90 s, washed twice with sterile distilled water, shaken in 6% (w/v) NaClO solution for 5 min, and washed 5–6 times with sterile distilled water (Koyama et al., 2012). The final washing occurred on LB medium and was cultured at 37 °C for 36 h to confirm that the root surface of cassava was completely sterilized. Surface sterilized samples were ground with sterilized with a mortar and pestle and inoculated on nitrogen-free semi-solid agar media with a grinding solution of 0.1 mL. After incubation at 28 °C for 5–7 days, the inoculates were transferred to fresh nitrogen-free media and incubated at 28 °C for 2 days. The transfer procedure was carried out three times to isolate a single strain.

### Microorganisms and growth conditions

The isolated strains were grown on an Ashby nitrogen-free agar plate (Sun et al., 2018) (0.2-g KH_2_PO_4_, 5-g CaCO_3_, 0.2-g MgSO_4_·7H_2_O, 10-g mannitol, 0.2-g NaCl, 15-g agar, 0.1-g CaSO_4_·2H_2_O per liter) and Döbereiner nitrogen-free agar plate (Luo et al., 2010) (5.0-g malic acid, 0.4-g KH_2_PO_4_·H_2_O, 0.2-g MgSO_4_·7H_2_O, 0.1-g NaCl, 0.02-g CaCl_2_·H_2_O, 0.01-g FeCl_3_, 0.002-g Na_2_MoO_4_·2H_2_O per liter; pH 7.0) at 28 °C for 5–7 days. All of the isolated strains were stored at −80 °C in LB containing 15% (v/v) glycerol for subsequent tests.

### Nitrogen fixation ability analysis of isolated strains

#### Evaluation of nitrogenase activity of isolated strains

Nitrogenase activity was detected by the acetylene reduction method (ARA) described by Rice et al. (1994) and Puri, Padda & Chanway (2018). A single strain was used to inoculate LB liquid medium and incubated in enriched culture for 24 h. Then, the suspension was used to inoculate 20-mL Döbereiner nitrogen-free liquid medium. The control treatment was not inoculated with nitrogen-free liquid medium, and the OD_600_ of bacterial suspension attained 0.6 at 30 °C and 160 r/min. An injector was used to draw out 5 mL of gas from the bottle, and 5 mL of high purity acetylene was injected into the bottle. After incubation at 30 °C and 160 r/min for 24 h, and 5 mL of gas was extracted to determine ethylene production by gas chromatography to determine the nitrogenase activity. To quantify ethylene production, the resulting chromatograms were used to integrate the area under the ethylene curve. The experiments were set up with four replicates for each treatment.

#### Evaluation of nitrogen fixation capacity of isolated strains

The nitrogen content in the culture medium was determined by the micro-Kjeldahl method to evaluate the nitrogen fixation effect of the isolated strains (Franche, Lindström & Elmerich, 2009; Rice et al., 1994; Puri, Padda & Chanway, 2018). The bacterial suspension was used to inoculate 50-mL Döbereiner nitrogen-free liquid culture medium and cultured for 7 days at 30 °C on 160 r/min. Subsequently, it was centrifuged for 10 min at 10,000 rpm. After digestion by sulfuric acid, the nitrogen content of the supernatant was determined by the micro-Kjeldahl method.

### Morphological and molecular identification of strain A02

#### Morphological identification

Strain A02 was cultured and purified on LB solid medium at 28 °C for 2 days. A single colony was obtained by the plate streak method. The morphological characteristics of the single colony were observed using a microscope.

#### Molecular identification

Isolated strain A02 was cultured in a 15-mL LB broth at 200 rpm at 30 °C. Genomic DNA of A02 was extracted using the CangWei century bacteria Gen DNA kit. The 16S rRNA gene of strain A02 was amplified using primers 27F (5′-AGAGTTTGATCMTGGCTCAG-3′) and 1492R (5′-GGTTACC TTGTTACGACTT-3′) (Weisburg et al., 1991). PCR conditions were as follows: initial denaturation for 2 min at 94 °C, 30 cycles of 30 s at 94 °C, 30 s at 55 °C and 30 s at 72 °C. The amplified fragments were recovered from agarose gel using the Universal DNA Purification kit (Tiangen, China) and sequenced by Sangon Biotech, Shanghai. The 16S rRNA gene sequences of A02 were matched with those from the NCBI BLAST search (https://www.ncbi.nlm.nih.gov/). The sequences of bacteria with high similarity to strain A02 were used for phylogenetic tree analysis by using the ClustalX and MEGA 5.0 software to identify bacterial attribution. The MUSCLE program of the integrated MEGA 5.0 software was used to run sequence combinations with default parameters, the neighbor-joining method was used to construct a phylogenetic tree, and the bootstrap method was used to calculate the tree branch node expansion value 1,000 times.

#### Effect of pH on nitrogen fixation activity of strain A02

The suspension was used to inoculate a nitrogen-free Döbereiner liquid medium. The pH of the medium was set to 5.0, 5.5, 6.0, 6.5, 7.0, 7.5, and 8.0. When the OD_600_ of the bacterial suspension reached 0.6 at 30 °C and 160 r/min, nitrogenase (acetylene reduction) activity was determined by the acetylene reduction method (ARA).

#### Effect of nitrogen on the nitrogen fixation activity of strain A02

The suspension was used to inoculate Döbereiner nitrogen-free liquid medium, and peptone was added to the medium as a nitrogen source. The nitrogen potency (peptone) was 0, 0.02, 0.04, 0.06, 0.08, 0.1, 0.12, 0.14, 0.16, 0.18, and 0.2 g L^−1^. Each treatment was repeated four times. When the OD_600_ of the bacterial suspension reached 0.6 at 30 °C on 160r/min, the activity of nitrogenase was determined by the acetylene reduction assay (ARA).

### Characterization of strain A02

#### Auxin activity test

The indole-3-acetic acid (IAA) produced by the cultures was estimated by growth in King B (KB) medium supplemented with l-tryptophan as a precursor of IAA (Ouyabe et al., 2020). Strain A02 was incubated in a 1-ml LB broth and obtained by centrifugation at a speed of 10,000 rpm for 5 min, with sterile water washing twice for 5 min. The bacterial suspension was diluted 10 times with sterile water, and a 200-uL suspension was used to inoculate 10-mL tryptophan growth medium. Cultures were incubated at 28 °C in the dark for 2 days. A 5-mL bacterial suspension was centrifuged for 5 min, then Salkowski coloring reagent (35% HClO_4_ 50 mL, 0.5 M FeCl_3_ 1 mL) was added, and the supernatant mixture at a ratio of 2:3 was incubated in the dark for 30 min at 28 °C. After the reaction, absorbance at 530 nm was estimated.

#### Phosphate-solubilizing activity test

Strain A02 was cultured for 2 days in LB broth to an OD_600_ of 0.6 and then transferred to the National Botanical Research Institute’s phosphate growth (NBRIP) broth (Yanlei & Xiaoping, 2018) and incubated at 28 °C and 160 rpm for 8 days. The culture supernatant was obtained by centrifugation at 10,000 rpm at 4 °C for 15 min. The ability of A02 to solubilize phosphate was tested using the molybdenum antimony anticolorimetric method (Yanlei & Xiaoping, 2018). The soluble phosphorus content was calculated by determining the absorbance at 700 nm. The pH value of the medium was measured using a pH meter. The content of titratable acid in the medium was determined by the neutralizing titration method with 0.1 mol L^−1^ NaOH.

#### Analysis of physiological and biochemical characteristics of strain A02

The colony, morphology, physiological, and biochemical characteristics of the strain were assessed according to the methods of the *Common Bacterial System Identification Manual*. Crystalline violet-saffron was used for Gram staining. Ink was used for capsule staining, and malachite green-saffron was used for spore staining. The physiological and biochemical characteristics were analyzed with methyl red (MR), indole, the Vogs–Proskauer (VP) test, amylolysis, gelatin liquefaction, citrate, dextrose acidogenesis, lactose, sucrose, and maltose.

#### N-fixing and plant growth promotion

The experiment was carried out at the Agricultural College of Guangxi University Farm Test Base from April to November 2019. The soil was strongly acid (pH 6.36). The amount of SOM, total N, available N, total P, available P, total K, and available K were 9.8 mg kg, 1.8508 g kg^−1^, 66.5 mg kg^−1^, 0.177 g kg^−1^, 33.6 mg kg^−1^, 3.17g kg^−1^ and 107.57 mg kg^−1^, respectively. Strain A02 was cultured in LB liquid medium to the logarithmic phase (OD_600_ = 0.6–0.8). The stems of cassava seed were soaked with a liquid bacterial treatment (treatment group) and LB liquid medium (control group) for 1 h before planting, and were planted in 6 rows, each with 12 plants. One month later, roots were irrigated with 45-mL A02 bacteria solution (treatment group) and LB culture solution (control group). On January 5, 2020, the roots, stems, and leaves of cassava were harvested, and their biomass and nitrogen content were measured.

### Statistical analysis

The data were statistically analyzed for univariate risk factors using SPSS 25.0 (SPSS Inc., Chicago, IL, USA). Mean values were compared using Duncan’s new multiple range test at a 5% (*P* < 0.05) level of significance between treatments.

## Results

### Isolation of endophytic bacteria from cassava roots and comparison of nitrogen fixation capacity

**Figure 1:**
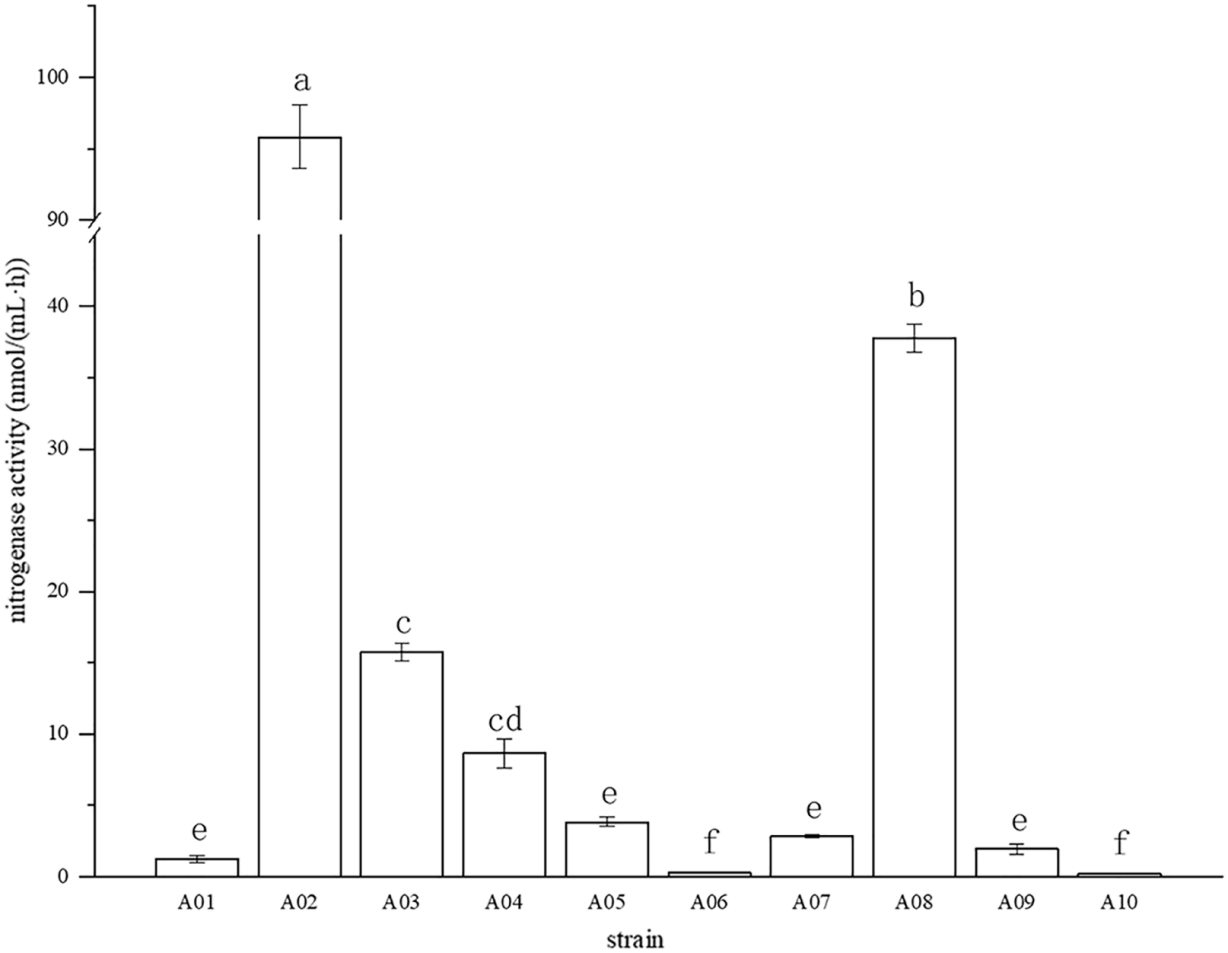
Nitrogenase activity in nitrogen-free liquid medium.

**Figure 2:**
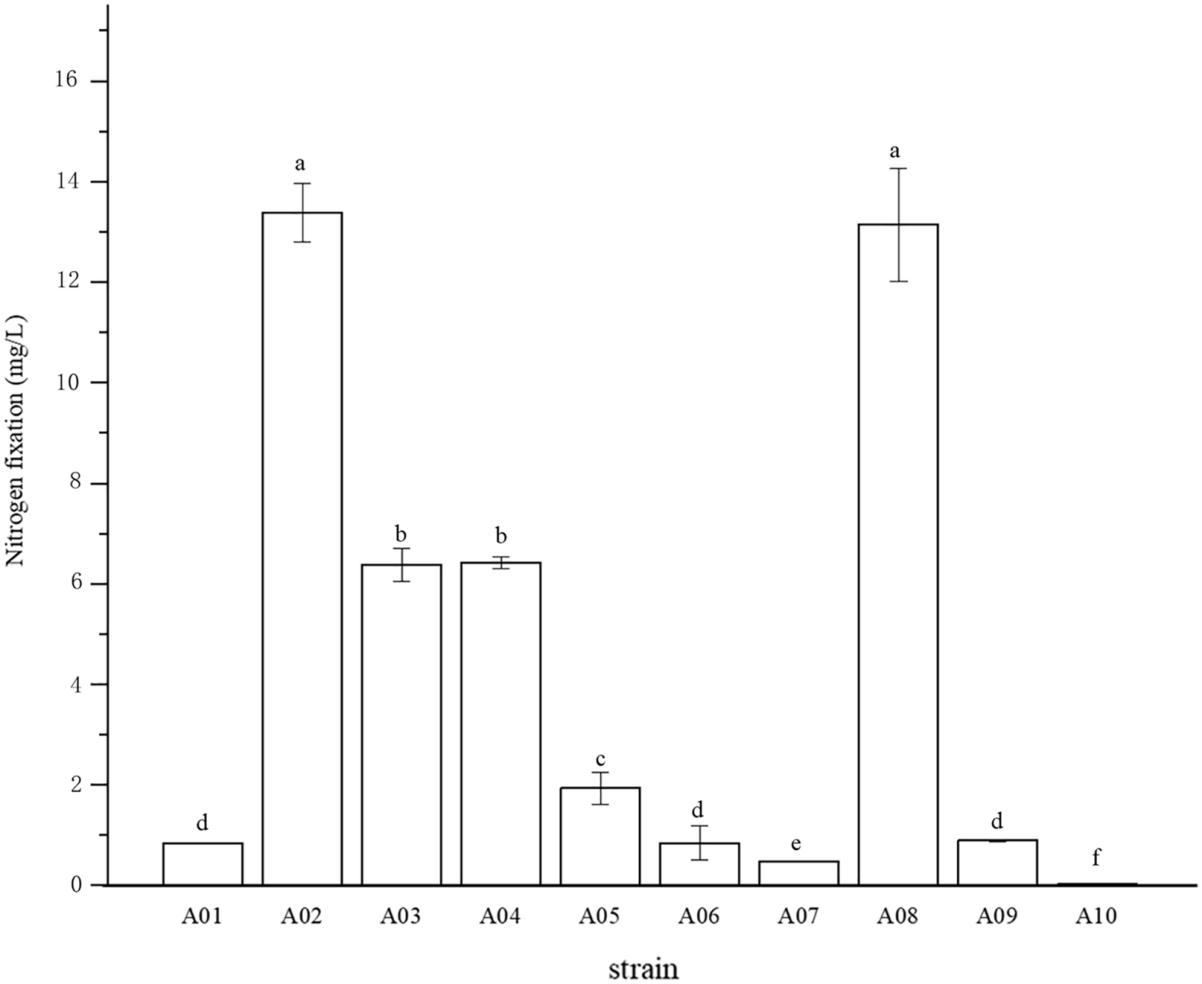
Nitrogen fixation in nitrogen-free liquid medium.

The root surface of cassava was thoroughly sterilized. It was concluded that the isolated strains were obtained from inside the fine roots of cassava. In this study, bacterial colonies appeared on the inoculated plates after 3 days of inoculation, while no colonies grew on the control plates. A total of 10 endophytic bacterial strains were isolated from the roots of cassava based on morphological characteristics, and the isolates were purified and stored at 4 °C. The 10 endophytic bacteria were named A01, A02, A03, A04, A05, A06, A07, A08, A09, and A10. The strains were selected for tube preservation and subsequent experiments. The nitrogenase activities of 10 strains are shown in Fig. 1. The highest nitrogenase activity of 95.81 nmol mL^−1^ h^−1^ was noted in strain A02, followed by strain A08. The activities of the other eight strains were all less than 20 nmol mL^−1^ h^−1^. Strain A06 and A10 had the lowest 0.29 and 0.24 nmol mL^−1^ h^−1^ of nitrogenase activity, indicating that the nitrogenase activities of different strains were significantly different. The biological nitrogen fixation activities of the 10 strains are shown in Fig. 2. Strain A02 had the strongest biological nitrogen fixation ability, followed by strain A08. After 7 days of culture, the nitrogen content in the medium was 13.38 mg L^−1^ and 13.14 mg L^−1^. Thus, strain A02 was selected for further analysis.

### Identification of strain A02

**Figure 3:**
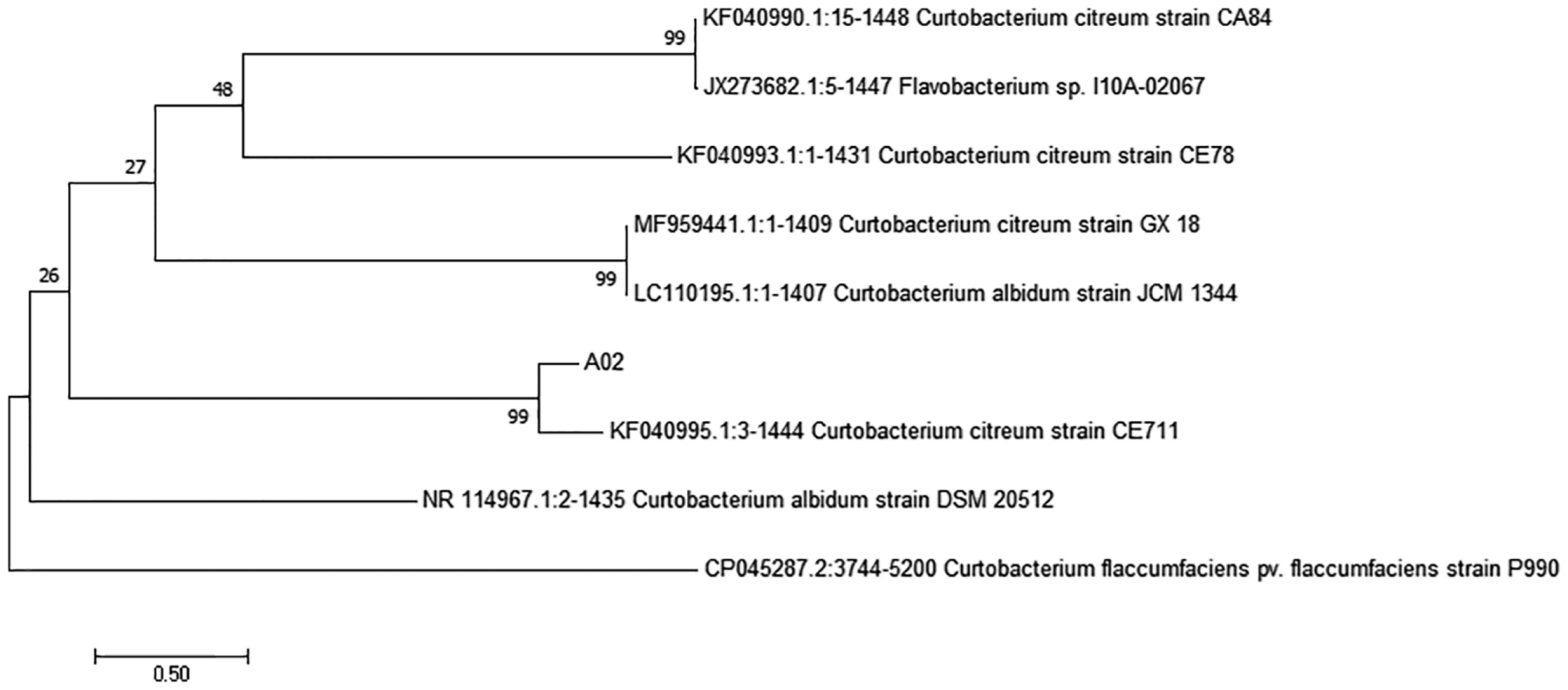
Phylogenetic tree based on 16S rRNA gene sequences of strain A02 from cassava roots. The tree was constructed by the neighbor-joining method using MEGA 7.0. The scale bar corresponds to 0.03 substitutions per nucleotide position. Numbers on the branches are bootstrap percentages. GenBank accession numbers are presented above.

**Figure 4:**
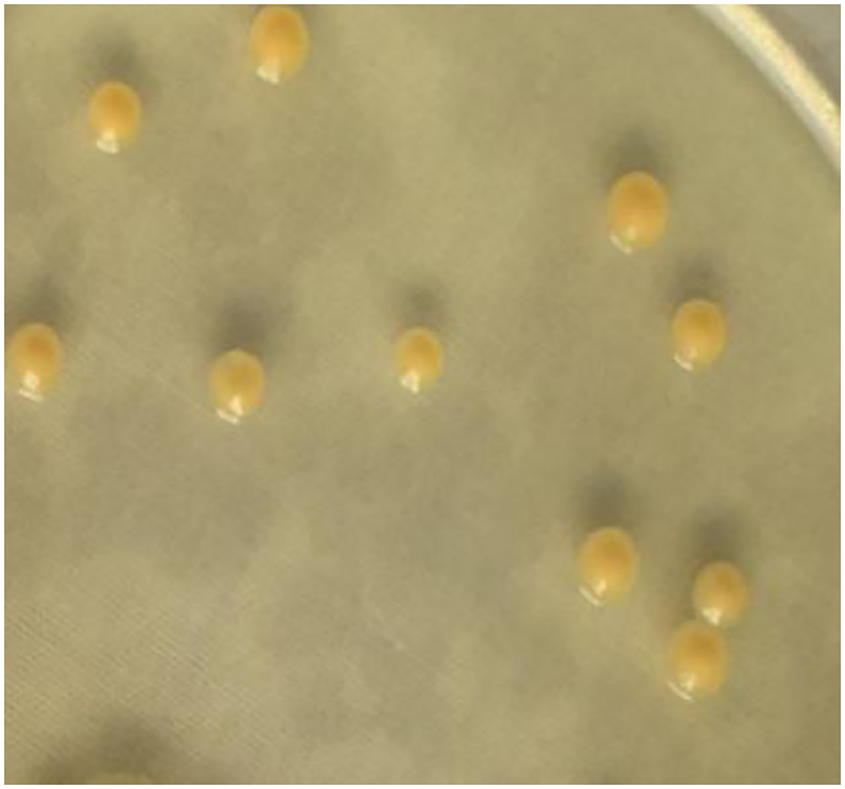
Colony morphology of strain A02 on an LB plate.

The 16S rRNA sequence of strain A02 was amplified by PCR. After sequencing, a 1500 bp rRNA sequence was obtained, and the GC content was 72.03%. Based on the BLAST search in NCBI, strain A02 showed 99.38% identity with *Curtobacterium citreum* CE711 (Fig. 3). Morphological analysis showed that strain A02 was rod-shaped and had no spores or capsule. Colonies of strain A02 were round, yellow, and non-transparent with a smooth surface and regular edges (Fig. 4). Strain A02 was identified as Actinobacteria *Curtobacterium citreum* by the General Microbial Center of China Microbial Species Preservation and Management Commission (CGMCC) and preserved (CGMCC Number: 12181). Based on the morphological characteristics, 16SrRNA gene sequence analysis, and CGMCC identification results, strain A02 was named *Curtobacterium* sp. A02, belonging to Actinobacteria, Actinobacterales, Microbacteriaceae, *and Curtobacterium*.

### Analysis of physiological and biochemical characteristics of strain A02

The physiological and biochemical characteristics of the A02 strain are shown in Table 1. Strain A02 was a Gram-positive bacterium. The glucose oxidative fermentation test and VP test were positive, but the MR test was negative. The gelatin liquefication test was negative, and the utilization test of starch, lactose, sucrose, maltose, and citric acid were also negative. Strain A02 is a gram-positive bacterium. The glucose oxidative fermentation test and VP test were positive, while the MR test was negative, indicating that A02 decomposed glucose into pyruvate and then further converted it into non-acidic end products, resulting in less organic acids. None of the five carbon sources (starch, lactose, sucrose, maltose, and citric acid) could be utilized, indicating that A02 had a narrow range of carbon sources, which might be related to the long-term survival adaptability of the A02 strain in cassava. A negative gelatin liquefaction test indicated that A02 could not secrete gelatinase and would not decompose protein after entering the plant cells to avoid adverse effects on their normal physiological activities.

**Table 1:** Physiological and biochemical characteristics of strain A02.

Physiological and biochemical characteristics	A02
Methyl red (MR)	−
Indole	+
VP	+
amylolysis	−
Gelatin liquefaction	−
Citrate	−
Dextrose acidogenesis	+
Lactose	−
Sucrose	−
Maltose	−
Gram stain	+
Capsule stain	−
Spore stain	−

**Note:**

“+” means that the physiological and biochemical test of this bacterium was positive, and “−” means that the physiological and biochemical test of this bacterium was negative.

A02 showed a positive reaction for IAA production by producing a pink to red color. The quantitative estimation of IAA at 530 nm was confirmed by ELISA. The highest IAA production was 1.56 mg mL^−1^. After 2 days of culture of the A02 strain, the solubility of soluble phosphorus in the medium reached 101.23 mg mL^−1^, the titratable acid content was 0.15 mg mL^−1^, and the pH decreased from 6.78 to 4.50 (Table 2). Therefore, it was concluded that the A02 strain had the ability to acidify the environment and dissolve insoluble phosphorus.

**Table 2:** Typical characteristics of phosphorus solubilizing ability.

Number	P solubilization (mg mL^−1^)	titratable acid (mg mL^−1^)	pH	IAA (mg mL^−1^)
CK	1.01 ± 0.14	0.01 ± 0.0002	6.78	0
A02	101.23 ± 1.43	0.15 ± 0.002	4.50	1.56

**Note:**

IAA stand for auxin is indole acetic acid.

### Optimizing growth conditions of strain A02

#### Effect of pH on the nitrogen fixation activity of strain A02

**Figure 5:**
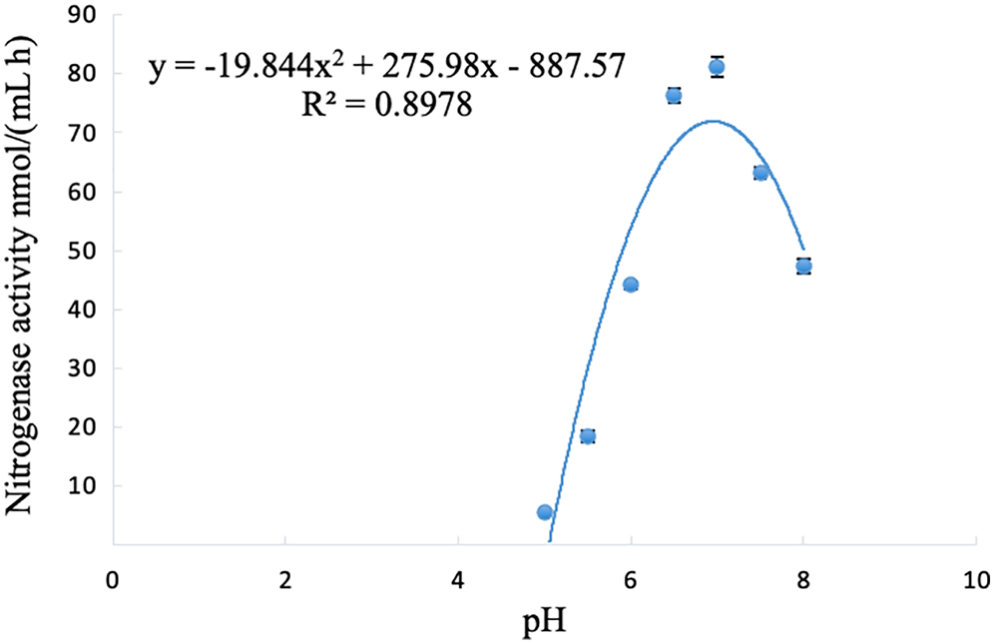
Influence of pH on the nitrogenase activity of strain A02.

The nitrogenase activity of strain A02 changed with the change in pH, and initially showed an increasing trend but then decreased with the increase in pH (5–8). The nitrogenase activity of strain A02 was the highest when the pH was 6.95 (Fig. 5).

#### Effect of nitrogen on the nitrogen fixation activity of strain A02

**Figure 6:**
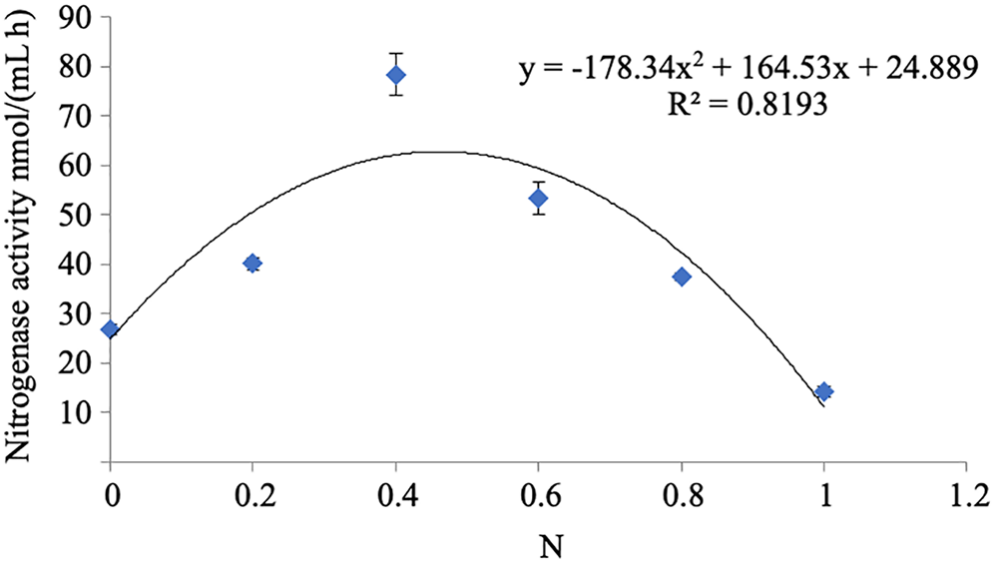
Influence of N on the nitrogenase activity of A02 strain.

The nitrogenase activity of strain A02 increased with the increase in the content of nitrogen in the range of 0–0.04 g L^−1^ and decreased with the increase in the content of nitrogen in the range of 0.04–0.1 g L^−1^ (Fig. 6). Therefore, nitrogen had a strong correlation with the nitrogenase activity of strain A02. Furthermore, the nitrogenase activity of strain A02 was highest when the nitrogen content in the medium was 0.05 g L^−1^.

#### N-fixation and plant growth promotion of strain A02

**Figure 7:**
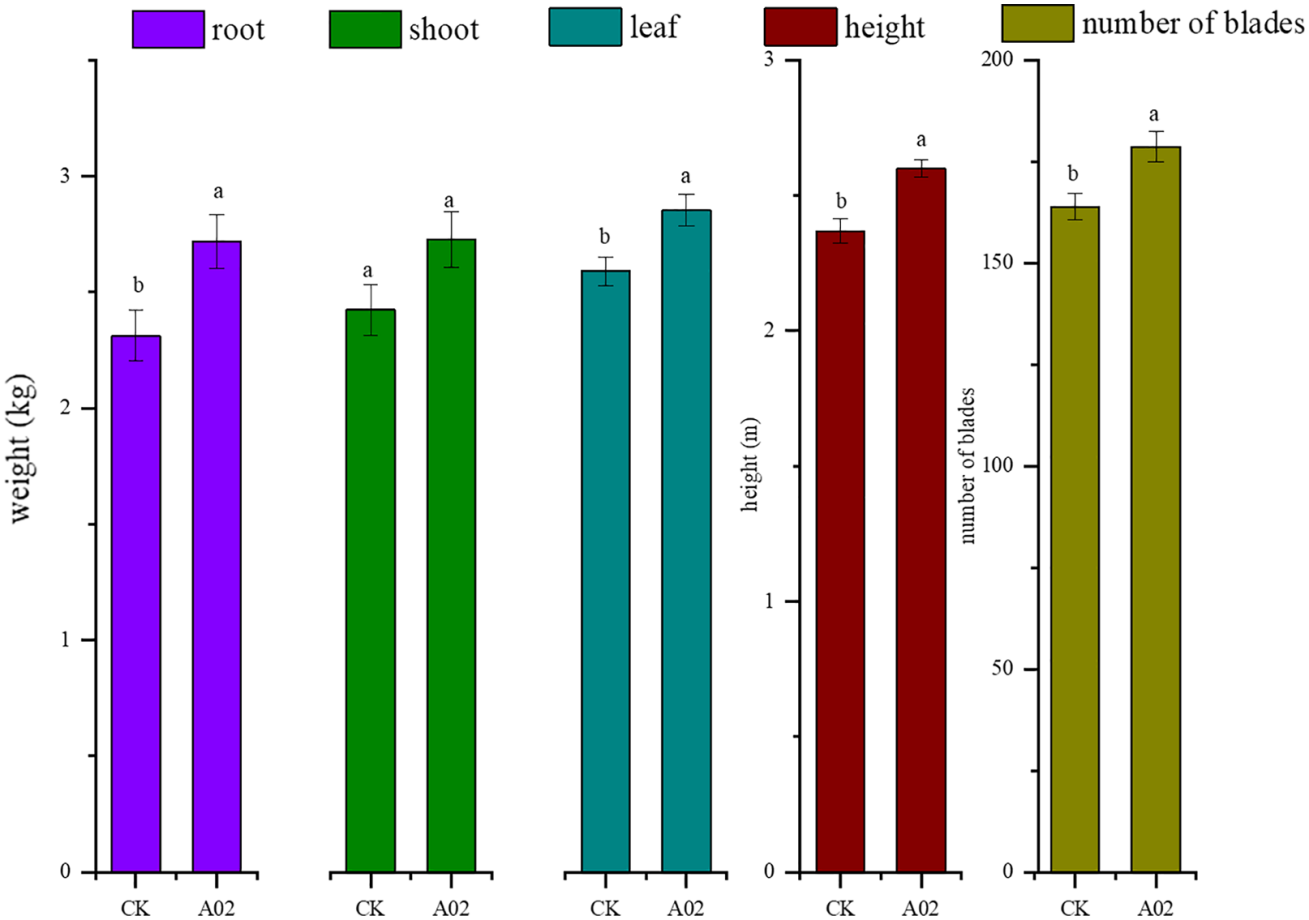
Effect of strain A02 on the growth of cassava.

Results showed that the biomass of roots, stems, and leaves of cassava inoculated with A02 significantly increased by 17.6%, 12.6%, and 10.3%, respectively, compared with that of the control (without A02 inoculation) (Table 3; Fig. 7). Based on 800 plants per acre, the tuber yield of cassava inoculated with A02 could be increased by 0.41 kg acre^−1^ with a 17.75% increase in yield.

**Table 3:** Nitrogenase activity and nitrogen content of cassava after inoculation with A02 and no nitrogen application in a field experiment.

Treatment	Nitrogenase activity (nmol mL^−1^ h^−1^)			Nitrogen content (mg g^−1^)		
	Leaves	Stems	Roots	Leaves	Stems	Roots
A02	97.97 ± 19.01a	46.17 ± 3.88a	58.3 ± 3.98a	47.01 ± 0.82a	14.84 ± 0.32a	13.48 ± 0.72a
-N	58.79 ± 3.63b	46.97 ± 5.11a	48.37 ± 3.17b	36.90 ± 0.81b	13.83 ± 0.39a	12.28 ± 0.60a

**Note:**

A02, treatment with A02 inoculation; -N, treatment without nitrogen.

## Discussion

Nitrogen plays an important role in cassava growth. A previous study reported that cassava had the ability to grow in low-fertile soil with the application of less fertilizer (Omondi et al., 2018). Endophytic nitrogen-fixing bacteria are colonized in plants and can effectively provide nitrogen to plants with no need to form specific nodules. Currently, there are few reports on endophytic bacteria in cassava (Reinhardt et al., 2008; de Barros Silva Leite et al., 2018). Several endophytic bacteria from cassava were isolated and identified as Achromobacter, Bacillus, Burkholderia, Enterobacter, Pantoea, and Pseudomonas spp.

In this study, 10 strains were isolated from cassava roots and grown on nitrogen-free medium. Overall, the higher 95.81 and 37.77 nmol mL^−1^ h^−1^ nitrogenase activities of strains A02 and A08 were noted, but the other eight strains had low nitrogenase activity. According to the morphological characteristics and 16S rRNA analysis of strain A02, it was classified as *Curtobacterium citreum* and named Curtobacterium sp. A02. *Curtobacterium citreum* was first isolated from Chinese rice (Mano et al., 2007) and has since been found in shy suckering chrysanthemum “Arka Swarna” (Panicker et al., 2007), strawberry fruit (Pereira et al., 2012), *Citrus sinensis* (Garrido et al., 2016), and sorghum (Bourles et al., 2019).

After 2 days of culture, the content of IAA in the medium of the A02 strain reached 1.56 mg mL^−1^, indicating that the A02 strain had the ability to produce and secrete IAA. *Pseudomonas aeruginosa* AL2-14B produced 114.79 μg mL^−1^ IAA, and Sinorhizobium fredii NGR234 produced 0.16 μmol mL^−1^ IAA (Roberto, Anna & Carmen, 2017). Enterobacter roggenkampii ED5 produced 732.93 μg mL^−1^ IAA (Jun et al., 2020). In comparison, strain A02 had a stronger ability to produce IAA. The solubility of soluble phosphorus in the medium reached 101.23 mg mL^−1^, the titratable acid content was 0.15 mg mL^−1^, and the pH decreased from 6.78 to 4.50, after 2 days of culture of strain A02 (Table 1). Therefore, it was concluded that strain A02 had the ability to acidify the environment and dissolve insoluble phosphorus. Fang et al. (2019) found that these secondary metabolites (IAA and soluble phosphorus) could promote plant growth (Fang et al., 2019). Combined with the results of glucose oxidation fermentation, VP, and MR tests, the phosphorus-dissolving ability of the A02 strain might be related to the secretion of organic acids.

The nitrogenase activity of soybean rhizobia reached 23,000 nmol mL^−1^ h^−1^ (Ma et al., 2020). As shown in Table 4, the nitrogenase activity of different plants combined with nitrogen-fixing bacteria greatly varies. The nitrogenase activity of sugarcane-associated nitrogen-fixing bacteria ranged from 65 to 3,187.8 nmol mL^−1^ h^−1^. The nitrogenase activity of maize and rice was about 100–200 nmol mL^−1^ h^−1^, while woody plants was 2.5 nmol mL^−1^ h^−1^. The nitrogen-fixing ability of cassava-associated nitrogen-fixing bacteria A02 was similar to that of maize- and rice-associated nitrogen-fixing bacteria.

**Table 4:** Nitrogenase activity of some endophytic nitrogen-fixing bacteria in the past 5 years.

Name	Nitrogenase activity (nmol mL^−1^ h^−1^)	Symbiosis plant	Author, Year Journal
*Pantoea agglomerans* XD20	3,187.8	sugarcane	(Mao et al., 2019) *Acta Botanica Boreali-Occidentalia Sinica*
*Pantoea sp*. NN08200	2,445	sugarcane	(Shi et al., 2019), *Microbiology China*
*Stenotrophomonas maltophilia* B11S	1,456.23	sugarcane	(Xing et al., 2016) *Sugar Tech*
*actinobacterial* WZS021	65	Sugarcane	(Wang et al., 2017) *Sugar Tech*
*Sphingomonas trueperi* NNA-14	350	Giant reed	(Xu et al., 2018) *Journal of Basic Microbiology*
*Klebsiella pneumoniae subspecies* 1′13	103.077	Guangxi wild rice	(Tan et al., 2017) *Chinese Journal of Applied and Environmental Biology*
*Devosia* RKZ210	2.98	highland barley	(Liu et al., 2017) *Journal of Triticeae Crops*
*K. radicincitans* GXGL-4A	232.94	Maize	(Li et al., 2016) Microbiology China
*Pseudomonas migulae*	2.8	American black pine	(Puri, Padda & Chanway, 2018) *Canadian journal of forest research*,
*Paenibacillus* L201	5,630	Bryophyllum pinnatum	(Liu et al., 2018) *Antonie Van Leeuwenhoek International journal of general and molecular microbiology*,

The contribution of tuber crops for the energy requirement of global population is 3.9%. Out of which 1.5% from sweet potato, 1.9% from cassava, and 0.3% from yams and other tuber crops (Birch et al., 2012). Further, similar to rhizobium, the associative nitrogen-fixing bacteria encode nitrogenase and utilize atmospheric nitrogen through biological nitrogen fixation (BNF) to convert N_2_ into inorganic nitrogen-containing compounds, such as ammonia (NH_3_), and improve the growth and yield of sugarcane (Matoso et al., 2021), sorghum (Rout & Chrzanowski, 2009), maize, wheat, cucumber (Yongbin et al., 2019). Our experiment confirmed that the nitrogenase activities, nitrogen contents and biomass of cassava seedlings with inoculation of strain A02 increased compared with that of the seedlings without inoculation. Combined with the results of the biochemical characteristics of A02, it was speculated that the promoting effect of strain A02 on cassava growth was not only due to the effect of biological nitrogen fixation but also due to other promoting effects of strain A02, such as the secretion of IAA and the ability of phosphorus dissolution. Furthermore, Curtobacterium was reported in some plants, *e.g*., *C. flaccumfaciens* strain ME1 significantly promoted cucumber growth and enhanced disease resistance (Raupach & Kloepper, 2000); Curtobacterium sp. NM1R1 infection promoted plant growth, increased the seed germination rate, and enhanced seedling tolerance to Zn (Román-Ponce et al., 2017); C. creum could reduce the migration of metal Ni from roots to the above-ground parts of sorghum but had no promoting effect on sorghum growth (Bourles et al., 2019). In addition to promoting growth, Curtobacterium also plays a certain role in improving heavy metal tolerance and disease resistance, which will be the next research direction for the A02 strain.

## Conclusion

In this study, we demonstrated the possibility of isolating associated nitrogen-fixing bacteria from cassava roots. Ten strains were screened by nitrogen-free medium, among which A02 had the highest nitrogenase activity. The results of bacterial morphological characteristics and 16S rRNA BLAST showed that the strain belonged to *Curtobacterium citreum*. The results of cassava tie-back showed that A02 increased the accumulation of nitrogen and promoted the growth of cassava, which was not only the result of biological nitrogen fixation but also related to the growth-promoting effects of IAA secretion and phosphorus dissolution. The results showed that the A02 strain had the potential to be a good candidate strain for promoting crop yield. To further explore this area and its application scope, future research is needed to study the effect of the A02 strain on the improvement of growth in other crops.

## Supplemental Information

10.7717/peerj.12677/supp-1 Supplemental Information 1 Typical characteristics of phosphorus solubilizing ability. * Each treatment with four replications, n = 4.

10.7717/peerj.12677/supp-2 Supplemental Information 2 Nitrogenase activity and nitrogen content of cassava after inoculation with A02 and no nitrogen application in a field experiment. * Each treatment with four replications, n = 4.

10.7717/peerj.12677/supp-3 Supplemental Information 3 Nitrogenase activity of some endophytic nitrogen-fixing bacteria in the past five years.

10.7717/peerj.12677/supp-4 Supplemental Information 4 Nitrogenase activity in nitrogen-free liquid medium. * Each treatment with four replications,n = 4.

10.7717/peerj.12677/supp-5 Supplemental Information 5 Nitrogen fixation in nitrogen-free liquid medium. * Each treatment with four replications, n = 4.

10.7717/peerj.12677/supp-6 Supplemental Information 6 Influence of pH on the nitrogenase activity of strain A02. * Each treatment with four replications, n = 4.

10.7717/peerj.12677/supp-7 Supplemental Information 7 Influence of N on the nitrogenase activity of the A02 strain. * Each treatment with four replications, n = 4.

10.7717/peerj.12677/supp-8 Supplemental Information 8 Effect of strain A02 on the growth of cassava. * 22 plants were measured for each treatment, n = 22

10.7717/peerj.12677/supp-9 Supplemental Information 9 Data of tables and figures.

## Additional Information and Declarations

### DNA Deposition

The following information was supplied regarding the deposition of DNA sequences: The sequence is available at GenBank: MW683362.1.

### Data Availability

The following information was supplied regarding data availability: The raw data is available in the Supplemental File and the sequence is available at NCBI: MW683362.
