# Supplementary material for: Isolation, identification and characterization of nitrogen fixing endophytic bacteria and their effects on cassava production
Source: PeerJ. 2022 Jan 25;10:e12677. doi: 10.7717/peerj.12677 (PMC8796710; doi:10.7717/peerj.12677)
Supplement: Supplemental Information 1 — * Each treatment with four replications, n = 4. [file peerj-10-12677-s001.docx]

Table 2 Typical characteristics of phosphorus solubilizing ability

| Number |  | Titratable acid (mg mL^-1^) | pH | IAA (mg mL^-1^) |
| --- | --- | --- | --- | --- |
| CK | 1.01±0.14 | 0.013±0.002 | 6.78 | 0 |
| A02 | 101.2±1.4 | 0.150±0.004 | 4.50 | 1.56±0.02 |

|  |  | 1 | 2 | 3 | 4 | Average | S.E. |
| --- | --- | --- | --- | --- | --- | --- | --- |
| P solubilization (mg mL^-1^) | CK | 1.103 | 0.842 | 1.355 | 0.75 | 1.01 | 0.14 |
|  | A02 | 102.85 | 103.01 | 102.05 | 97.02 | 101.2 | 1.4 |
| titratable acid  (mg mL^-1^) | CK | 0.015 | 0.015 | 0.016 | 0.007 | 0.013 | 0.002 |
|  | A02 | 0.150 | 0.154 | 0.156 | 0.140 | 0.150 | 0.004 |
| pH | CK | 6.11 | 7.19 | 6.95 | 6.85 |  |  |
|  | A02 | 4.5 | 4.47 | 4.48 | 4.56 |  |  |
| IAA  (mg mL^-1^) | CK | 0 | 0 | 0 | 0 | 0 | 0 |
|  | A02 | 1.619 | 1.506 | 1.555 | 1.562 | 1.560 | 0.02 |

* Each treatment with four replications, n=4.
