# Supplementary material for: Isolation, identification and characterization of nitrogen fixing endophytic bacteria and their effects on cassava production
Source: PeerJ. 2022 Jan 25;10:e12677. doi: 10.7717/peerj.12677 (PMC8796710; doi:10.7717/peerj.12677)
Supplement: Supplemental Information 2 — * Each treatment with four replications, n = 4. [file peerj-10-12677-s002.docx]

Table 3 Nitrogenase activity and nitrogen content of cassava after inoculation with A02 and no nitrogen application in a field experiment

| Treatment | Nitrogenase activity  (nmol mL^-1^ h^-1^) | | | Nitrogen content  (mg g^-1^) | | |
| --- | --- | --- | --- | --- | --- | --- |
|  | Leaves | Stems | Roots | Leaves | Stems | Roots |
| A02 | 97.97 ± 19.01a | 46.17 ± 3.88a | 58.3 ± 3.98a | 47.01 ± 0.82a | 14.84 ± 0.32a | 13.48 ± 0.72a |
| -N | 58.79 ± 3.63b | 46.97 ± 5.11a | 48.37 ± 3.17b | 36.90 ± 0.81b | 13.83 ± 0.39a | 12.28 ± 0.60a |

|  |  | Nitrogenase activity (nmol mL^-1^ h^-1^) | | | | | |
| --- | --- | --- | --- | --- | --- | --- | --- |
|  |  | 1 | 2 | 3 | 4 | Average | S.E. |
| Leaf | A02 | 110.50 | 117.58 | 78.28 | 85.53 | 97.97 | 9.50 |
|  | -N | 60.56 | 54.89 | 62.93 | 56.78 | 58.79 | 1.81 |
| Stem | A02 | 44.69 | 50.88 | 47.36 | 41.76 | 46.17 | 1.94 |
|  | -N | 40.30 | 46.47 | 48.55 | 52.56 | 46.97 | 2.56 |
| Root | A02 | 54.16 | 63.11 | 56.20 | 60.02 | 58.37 | 1.99 |
|  | -N | 45.45 | 46.85 | 52.77 | 48.40 | 48.37 | 1.59 |

* Each treatment with four replications, n=4.

|  |  | Nitrogen content (mg g^-1^) | | | | | |
| --- | --- | --- | --- | --- | --- | --- | --- |
|  |  | 1 | 2 | 3 | 4 | Average | S.E. |
| Leaf | A02 | 48.70 | 48.07 | 45.32 | 45.94 | 47.01 | 0.82 |
|  | -N | 34.95 | 37.63 | 36.35 | 38.69 | 36.90 | 0.81 |
| Stem | A02 | 14.68 | 14.58 | 14.59 | 15.50 | 13.83 | 0.32 |
|  | -N | 14.99 | 13.32 | 13.51 | 13.50 | 13.83 | 0.39 |
| Root | A02 | 15.76 | 13.68 | 11.78 | 12.71 | 13.48 | 0.72 |
|  | -N | 14.08 | 12.39 | 11.59 | 11.08 | 12.28 | 0.60 |

* Each treatment with four replications,n=4.
