# Supplementary material for: Isolation, identification and characterization of nitrogen fixing endophytic bacteria and their effects on cassava production
Source: PeerJ. 2022 Jan 25;10:e12677. doi: 10.7717/peerj.12677 (PMC8796710; doi:10.7717/peerj.12677)
Supplement: Supplemental Information 3 [file peerj-10-12677-s003.docx]

Table 4 Nitrogenase activity of some endophytic nitrogen-fixing bacteria in the past five years

| Name | nitrogenase activity  (nmol mL^-1^ h^-1^) | Symbiosis  plant | Author, Year  Journal |
| --- | --- | --- | --- |
| *Pantoea agglomerans* XD20 | 3187.8 | sugarcane | ([Mao et al., 2019](#_ENREF_34))  *Acta Botanica Boreali-Occidentalia Sinica* |
| *Pantoea sp.* NN08200 | 2445 | sugarcane | ([Shi et al., 2019](#_ENREF_53))*,*  *Microbiology China* |
| *Stenotrophomonas maltophilia* B11S | 1456.23 | sugarcane | ([Xing et al., 2016](#_ENREF_63))  *Sugar Tech* |
| *actinobacterial* WZS021 | 65 | Sugarcane | ([Wang et al., 2017](#_ENREF_61))  *Sugar Tech* |
| *Sphingomonas trueperi* NNA‐14 | 350 | Giant reed | ([Xu et al., 2018](#_ENREF_64))  *Journal of Basic Microbiology* |
| *Klebsiella pneumoniae subspecies*  1′13 | 103.077 | Guangxi wild rice | ([Tan et al., 2017](#_ENREF_58)) *Chinese Journal of Applied and Environmental Biology* |
| *Devosia* RKZ210 | 2.98 | highland barley | ([Liu et al., 2017](#_ENREF_26)) *Journal of Triticeae* *Crops* |
| *K. radicincitans* GXGL-4A | 232.94 | Maize | ([Li et al., 2016](#_ENREF_21)) Microbiology China |
| *Pseudomonas migulae* | 2.8 | American black pine | ([Puri et al., 2018](#_ENREF_42))  *Canadian journal of forest research*, |
| *Paenibacillus* L201 | 5630 | [Bryophyllum pinnatum](../../../ä) | ([Liu et al., 2018](#_ENREF_25))  *Antonie Van Leeuwenhoek International journal of general and molecular microbiology* |

Mao L, Huang C, Zhu K, Yang L, Li Y, Xing Y. 2019. Effect of Nitrogen-Fixing Strain XD20on the Growth of Different Sugarcane Varieties. Acta Botanica Boreali-Occidentalia Sinica 39(1):140-148.

Shi G, Zeng Q, Nong Z, Ye X, Cen Z, Li Y, Hu C. 2019. Identification of an endophytic nitrogen-fixing bacterium NN08200 from sugarcane and its growth promotion of sugarcane. Microbiology China 46(6): 1336-1345. doi: 10.13344/j.microbiol.china.180523.

Xing Y-X, Wei C-Y, Mo Y, Yang L-T, Huang S-L, Li Y-R. 2016. Nitrogen-Fixing and Plant Growth-Promoting Ability of Two Endophytic Bacterial Strains Isolated from Sugarcane Stalks. Sugar Tech 18(4): 373-379. doi: 10.1007/s12355-015-0397-7.

Wang Z, Solanki MK, Pang F, Singh RK, Yang L-T, Li Y-R, Li H-B, Zhu K, Xing Y-X 2017. Identification and Efficiency of a Nitrogen-fixing Endophytic Actinobacterial Strain from Sugarcane. Sugar Tech 19(5), 492-500. doi: 10.1007/s12355-016-0498-y.

Xu J, Kloepper JW, Huang P, McInroy JA, Hu CH. 2018. Isolation and characterization of N-2-fixing bacteria from giant reed and switchgrass for plant growth promotion and nutrient uptake. Journal of Basic Microbiology 58(5):459-471. doi: 10.1002/jobm.201700535.

Tan Z, Tan Z, Huang H, Zhang X, Liu L, Peng G. 2017. Isolation and phylogenetic analysis of endophytic nitrogen-fixing bacteria from Oryza officinalis in Wuxian. Chinese Journal of Applied and Environmental Biology 23(4):622-627.

Liu Z, Yue X, Wang B, Xue B, Wang Q. 2017. Diversity of Culturable Endophytic Nitrogen-Fixing Bacteria in Naked Barley Roots from Different Production Regions. Journal of Triticeae Crops 37(4): 565-569.

Li Q, Cheng J, Sun S, Chen Y. 2016. Isolation, identification and characterization of associative nitrogen-fixing endophytic bacterium Kosakonia radicincitans GXGL-4A in maize. Microbiology China 43(11):2456-2463. doi: 10.13344/j.microbiol.china.151071.

Puri A, Padda KP, Chanway CP. 2018. Evidence of endophytic diazotrophic bacteria in lodgepole pine and hybrid white spruce trees growing in soils with different nutrient statuses in the West Chilcotin region of British Columbia, Canada. Forest Ecology and Management 430:558-565. doi: 10.1016/j.foreco.2018.08.049.

Liu L, Yuan T, Yang F, Liu Z, Yang M, Peng G, Tan Z. 2018. Paenibacillus bryophyllum sp. nov., a nitrogen-fixing species isolated from Bryophyllum pinnatum. Antonie Van Leeuwenhoek International Journal of General and Molecular Microbiology 111(12):2267-2273. doi: 10.1007/s10482-018-1117-6.
