# Supplementary material for: Isolation, identification and characterization of nitrogen fixing endophytic bacteria and their effects on cassava production
Source: PeerJ. 2022 Jan 25;10:e12677. doi: 10.7717/peerj.12677 (PMC8796710; doi:10.7717/peerj.12677)
Supplement: Supplemental Information 4 — * Each treatment with four replications,n = 4. [file peerj-10-12677-s004.pdf]

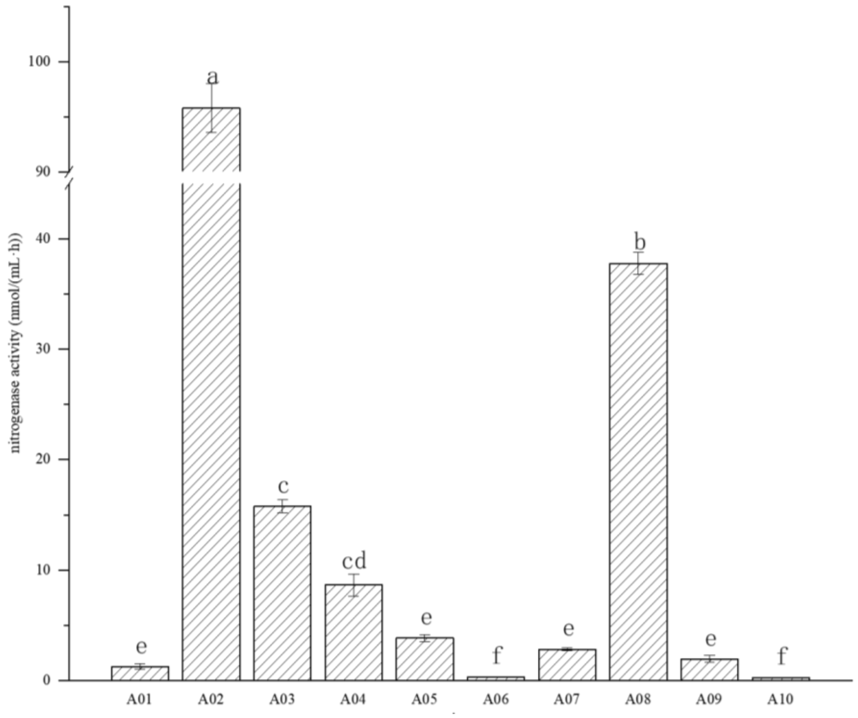

Figure 1 Nitrogenase activity in nitrogen-free liquid medium.

	Nitrogenase activity (nmol/(mL.h))					
	1	2	3	4	Average	S.E.
A01	1.795	0.584	1.415	1.181	1.24	0.25
A02	91.929	92.008	100.381	98.913	95.81	2.24
A03	17.423	14.763	15.144	15.658	15.75	0.59
A04	7.039	8.157	7.840	11.507	8.64	0.99
A05	3.641	4.532	3.014	4.152	3.83	0.33
A06	0.234	0.382	0.301	0.244	0.29	0.03
A07	2.885	2.730	3.074	2.581	2.82	0.11
A08	40.580	37.020	35.863	37.632	37.77	1.00
A09	2.328	1.872	2.518	1.026	1.94	0.33
A10	0.269	0.267	0.235	0.185	0.24	0.02

* Each treatment with four replications,n=4.
