# Supplementary material for: Isolation, identification and characterization of nitrogen fixing endophytic bacteria and their effects on cassava production
Source: PeerJ. 2022 Jan 25;10:e12677. doi: 10.7717/peerj.12677 (PMC8796710; doi:10.7717/peerj.12677)
Supplement: Supplemental Information 5 — * Each treatment with four replications, n = 4. [file peerj-10-12677-s005.pdf]

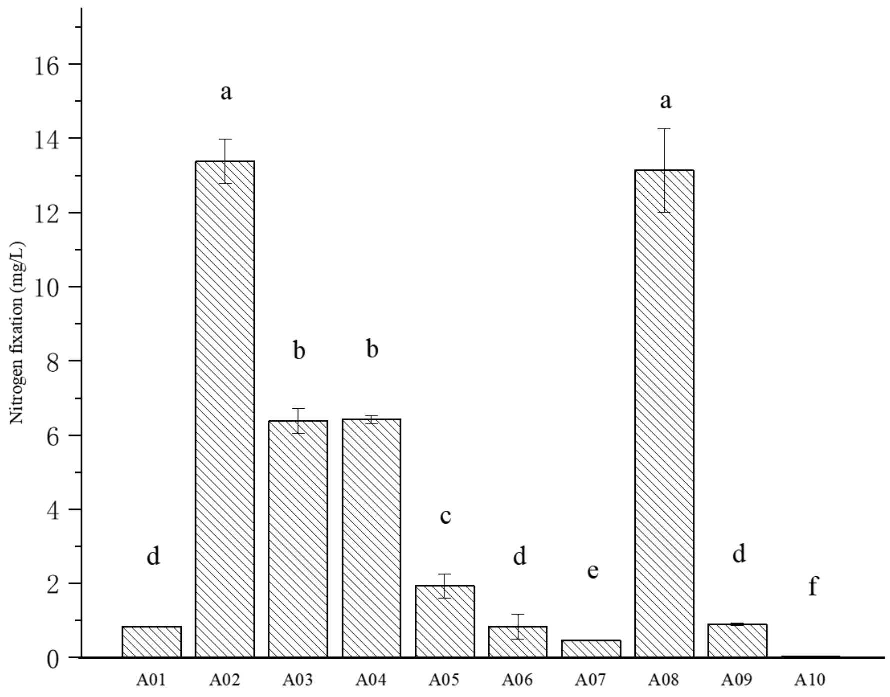

Figure 2 Nitrogen fixation in nitrogen-free liquid medium

	Nitrogen fixation (mg/L)					
	1	2	3	4	average	S.E.
A01	0.801	0.851	0.804	0.886	0.835	0.020
A02	14.88	12.86	13.68	12.13	13.38	0.591
A03	6.565	6.379	5.488	7.089	6.380	0.333
A04	6.965	6.315	6.213	6.181	6.419	0.184
A05	1.534	2.035	2.787	1.363	1.930	0.319
A06	1.045	1.702	0.301	0.318	0.842	0.335
A07	0.455	0.419	0.586	0.428	0.472	0.039
A08	15.79	12.90	10.29	13.60	13.14	1.134
A09	0.881	0.985	0.855	0.826	0.887	0.035
A10	0.046	0.012	0.010	0.029	0.024	0.008

* Each treatment with four replications, n=4.
