# Supplementary material for: Isolation, identification and characterization of nitrogen fixing endophytic bacteria and their effects on cassava production
Source: PeerJ. 2022 Jan 25;10:e12677. doi: 10.7717/peerj.12677 (PMC8796710; doi:10.7717/peerj.12677)
Supplement: Supplemental Information 6 — * Each treatment with four replications, n = 4. [file peerj-10-12677-s006.pdf]

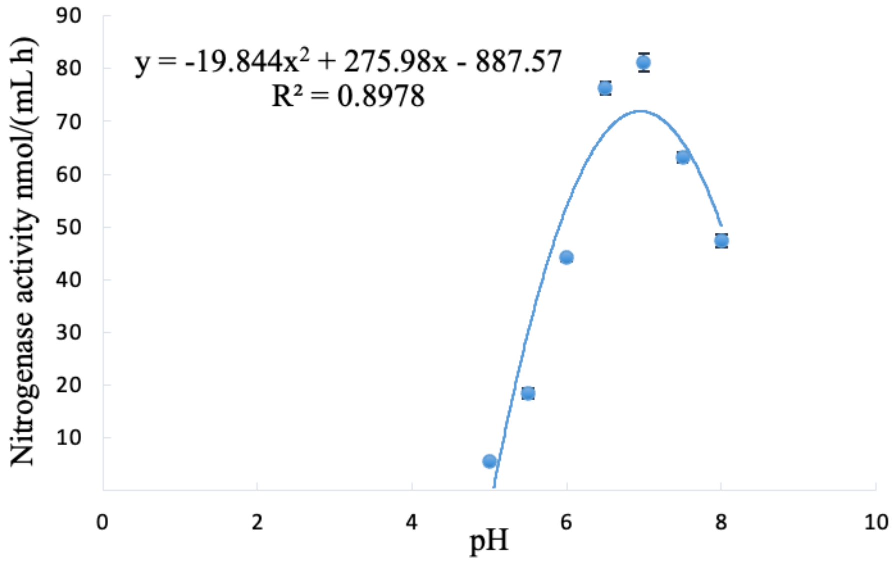

Figure 5 Influence of pH on the nitrogenase activity of strain A02

	Nitrogenase activity (nmol/(mL.h))			
pH	1	2	3	4
5	5.74	5.76	5.64	5.11
5	15.22	20.94	18.36	18.94
6	42.44	45.92	43.95	43.93
6.5	77.98	78.95	71.87	76.24
7	79.36	89.91	81.73	73.94
7.5	60.41	65.98	63.17	63.12
8	50.05	46.12	52.11	40.91

* Each treatment with four replications, n=4.
