# Supplementary material for: Isolation, identification and characterization of nitrogen fixing endophytic bacteria and their effects on cassava production
Source: PeerJ. 2022 Jan 25;10:e12677. doi: 10.7717/peerj.12677 (PMC8796710; doi:10.7717/peerj.12677)
Supplement: Supplemental Information 7 — * Each treatment with four replications, n = 4. [file peerj-10-12677-s007.pdf]

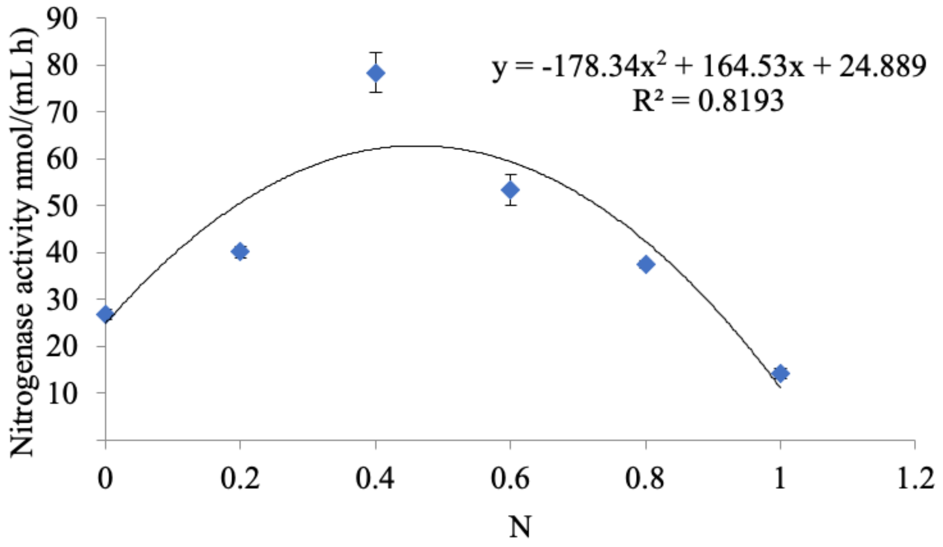

Figure 6 Influence of N on the nitrogenase activity of the A02 strain

	Nitrogenase activity (nmol/ (mL h)				
N ($g\ L^{-1}$)	1	2	3	4	Average
0	26.53	34.00	30.26	27.37	29.54
0.2	46.98	39.98	37.49	36.23	40.17
0.4	60.93	66.99	91.94	93.89	78.44
0.6	60.84	67.38	39.28	45.93	53.36
0.8	41.34	33.95	38.49	36.40	37.54
1.0	11.94	19.39	9.99	15.39	14.18

* Each treatment with four replications, n=4.
