# Supplementary material for: Isolation, identification and characterization of nitrogen fixing endophytic bacteria and their effects on cassava production
Source: PeerJ. 2022 Jan 25;10:e12677. doi: 10.7717/peerj.12677 (PMC8796710; doi:10.7717/peerj.12677)
Supplement: Supplemental Information 8 — * 22 plants were measured for each treatment, n = 22 [file peerj-10-12677-s008.pdf]

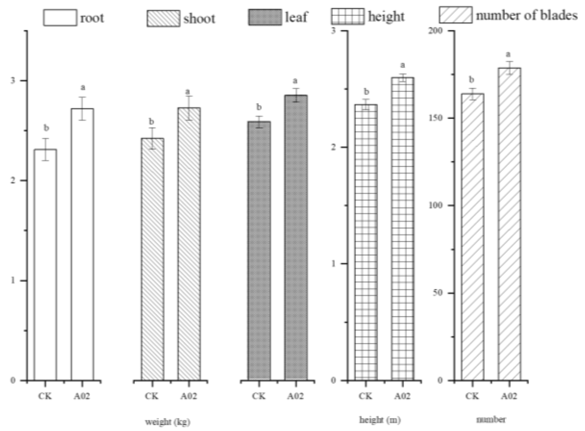


Figure 7 Effect of strain A02 on the growth of cassava

| | Leaf FW (kg plant$^{-1}$) | | Leaf FW (kg plant$^{-1}$) | | Leaf FW (kg plant$^{-1}$) | | Height (m) | |
|---|---|---|---|---|---|---|---|---|
| Plant number | Ck | A02 | Ck | A02 | Ck | A02 | Ck | A02 |
| 1 | 2.82 | 1.72 | 1.65 | 1.01 | 0.589 | 0.487 | 2.71 | 2.35 |
| 2 | 2.48 | 3.68 | 1.39 | 1.73 | 0.550 | 0.674 | 2.49 | 2.70 |
| 3 | 2.70 | 2.29 | 1.43 | 1.27 | 0.563 | 0.584 | 2.55 | 2.51 |
| 4 | 2.51 | 3.07 | 1.10 | 1.35 | 0.530 | 0.629 | 2.32 | 2.70 |
| 5 | 1.81 | 2.77 | 0.95 | 1.20 | 0.467 | 0.622 | 2.20 | 2.68 |
| 6 | 3.42 | 2.37 | 1.40 | 1.63 | 0.556 | 0.619 | 2.55 | 2.55 |
| 7 | 1.37 | 3.47 | 0.86 | 1.82 | 0.493 | 0.763 | 2.06 | 2.94 |
| 8 | 1.76 | 2.51 | 0.71 | 1.13 | 0.507 | 0.622 | 2.10 | 2.50 |
| 9 | 3.08 | 2.30 | 1.44 | 0.99 | 0.619 | 0.529 | 2.40 | 2.35 |
| 10 | 2.16 | 2.26 | 1.10 | 1.24 | 0.594 | 0.568 | 2.30 | 2.48 |
| 11 | 1.88 | 2.09 | 0.93 | 0.94 | 0.561 | 0.601 | 2.10 | 2.40 |
| 12 | 1.95 | 2.30 | 1.29 | 1.65 | 0.641 | 0.658 | 2.40 | 2.55 |
| 13 | 2.18 | 2.66 | 1.11 | 1.31 | 0.526 | 0.686 | 2.25 | 2.60 |
| 14 | 2.21 | 1.89 | 1.06 | 0.98 | 0.602 | 0.620 | 2.40 | 2.40 |
| 15 | 1.97 | 3.39 | 1.05 | 1.57 | 0.573 | 0.823 | 2.20 | 2.81 |
| 16 | 3.29 | 3.46 | 1.64 | 2.02 | 0.696 | 0.800 | 2.60 | 2.88 |
| 17 | 2.42 | 2.18 | 1.33 | 1.05 | 0.616 | 0.647 | 2.50 | 2.45 |
| 18 | 2.63 | 3.07 | 1.52 | 1.33 | 0.714 | 0.678 | 2.70 | 2.60 |
| 19 | 1.59 | 3.43 | 0.87 | 1.58 | 0.603 | 0.655 | 2.20 | 2.65 |
| 20 | 2.44 | 3.00 | 1.33 | 1.26 | 0.646 | 0.670 | 2.30 | 2.62 |
| 21 | 2.03 | 2.53 | 1.22 | 1.17 | 0.642 | 0.655 | 2.40 | 2.59 |
| 22 | 1.72 | 2.34 | 0.93 | 1.25 | 0.528 | 0.645 | 1.86 | 2.62 |
| 23 | 2.16 | 3.26 | 1.21 | 1.58 | 0.704 | 0.728 | 2.60 | 2.75 |
| 24 | 2.88 | 3.20 | 1.55 | 1.67 | 0.685 | 0.688 | 2.62 | 2.67 |
| Average | 2.31 | 2.72 | 1.21 | 1.36 | 0.592 | 0.652 | 2.37 | 2.60 |
| S.E. | 0.47 | 0.55 | 0.25 | 0.28 | 0.121 | 0.133 | 0.48 | 0.53 |

* 22 plants were measured for each treatment, n=22
